# The Incidence of Thyroid Cancer in Bethesda III Thyroid Nodules: A Retrospective Analysis at a Single Endocrine Surgery Center

**DOI:** 10.3390/diagnostics14101026

**Published:** 2024-05-16

**Authors:** Iyad Hassan, Lina Hassan, Nahed Balalaa, Mohamad Askar, Hussa Alshehhi, Mohamad Almarzooqi

**Affiliations:** 1 Department of Surgery, Burjeel Hospital, Abu Dhabi 7400, United Arab Emirates; lapsim1970@yahoo.de (L.H.); mohammad.askar@burjeel.com (M.A.); hussa.alshehhi@burjeelmedicalcity.com (H.A.); mohamed.almarzooqi@burjeel.com (M.A.); 2 Department of Surgery, Shaikh Shakhboot Medical City, Abu Dhabi 7400, United Arab Emirates; nbalalaa@ssmc.ae

**Keywords:** FNAC, Bethesda classification, thyroid cancer, indeterminate nodules, thyroid nodules, ultrasound-guided biopsy, thyroidectomy

## Abstract

Background: Fine-needle aspiration cytology (FNAC) is widely used to diagnose and monitor thyroid nodules. The Bethesda System for Reporting Thyroid Cytopathology (TBSRTC) is the standard for interpreting FNAC specimens. The risk of malignancy in Bethesda III nodules, also known as Atypia of Undetermined Significance (AUS), varies significantly throughout several studies published worldwide. This retrospective study examines the risk of cancer in thyroid FNAC categorized as Bethesda III as identified in the final histopathology of thyroidectomy specimens at a single endocrine surgery center. Methods: This retrospective cohort analysis included 1038 consecutive patients who underwent elective thyroid surgery with complete follow-up data between January 2020 and March 2024. Preoperative data on clinical and pathological characteristics have been collected. The final histopathology report from the thyroidectomy specimen was compared to the results of the preoperative FNAC on nodules that were judged to be Bethesda category III. Statistical methods were performed using SPSS version 29. Results: A total of 670 ultrasound-guided FNACs (64.5%) performed during the study period were included in the final analysis. The study population was predominantly female, represented by 79.6% of patients with a mean age of 42.5 (SD 12.1), while 20.4% were male and significantly older with mean age of 45.13 years (*p* = 0.02). The FNAC inadequacy rate was 5.1%, which was associated with a high risk of malignancy (6 out of 34; 17.6%). Out of the total sample size of 170 patients classified as group III, 57 were found to have malignancies in final surgical histopathology, representing 33.5% of the cases within this category. The secondary gender-related outcome analysis showed that female patients classified under the Bethesda II category had a significantly higher risk of malignancy, with a rate of 21.2%, compared to males who had a malignancy rate of 3.4% in the same Bethesda category (*p* = 0.001, chi-square test). However, the female patients exhibited prognostically superior non-invasive tumors compared to male individuals (*p* = 0.02, chi-square test). Conclusion: This study’s results indicate that Bethesda categories II and III are associated with a higher risk of malignancy in comparison to the reports of the first and third editions of the TBSRTC, particularly for female patients classified under category II.

## 1. Introduction

Thyroid nodules are common and carry a potential risk of cancer. In light of the increasing global prevalence of thyroid carcinoma in recent decades, evaluating thyroid nodules for malignancy has become an essential aspect of its medical management [1,2]. While environmental and genetic etiologies have been proposed to explain these rising patterns, a growing body of research suggests that improved healthcare availability and the use of advanced diagnostic technologies are the main causes of increases in the diagnosis of thyroid cancer [3]. Nevertheless, other authors have discussed a genuine rise in the occurrence of thyroid cancer (TC), along with a shift in its mortality rate. However, analyses of mortality rates specific to papillary thyroid cancer in the US have shown an annual rise in mortality of 1.1% overall and 2.9% specifically for papillary thyroid cancer between 1994 and 2013 [4]. Furthermore, a rising body of evidence indicates that the COVID-19 pandemic might have increased the aggressiveness of papillary thyroid cancer. As a result, it is essential to devote more attention to the comprehensive evaluation of thyroid nodule patterns [5,6]. Therefore, fine needle aspiration cytology is a widely used technique for the initial evaluation of thyroid nodules, and the Bethesda System for Reporting Thyroid Cytopathology is commonly used to categorize the results of FNAC [7,8]. Although fine-needle aspiration cytology (FNAC) is a valuable diagnostic instrument, it presents a number of technical challenges, including sample adequacy; therefore, representative samples must be obtained using the correct technique [9]. Nevertheless, patient cooperation, needle size, and operator expertise can all impact the sufficiency of the obtained sample. Additional obstacles in thyroid FNAC include the location and accessibility of nodules, blood interference, and cellular degeneration, which can be caused by prolonged aspiration or improper sample processing. Cellular degeneration compromises the quality of the specimen and subsequent interpretation. At last, FNAC specimens are subjectively interpreted, and cytopathologists may differ in their assessments. To tackle these technical obstacles, operator experience, the procedural technique, and the implementation of supplementary measures like on-site assessment and ancillary testing are crucial for optimizing the diagnostic yield and accuracy of FNAC in the context of thyroid nodules [10].

The Bethesda System for Reporting Thyroid Cytopathology (TBSRTC), third edition, made significant adjustments to improve thyroid nodule reporting and treatment. The nomenclature in this revised version was changed to clarify and standardize cytological results, minimizing ambiguity and promoting uniform reporting across institutions. Also, the risk stratification approach was modified to better predict thyroid nodule malignancy based on cytology. This helped physicians make better-informed choices about patient care and follow-ups [11].

However, thyroid nodules present a significant clinical challenge due to the need to distinguish between benign and malignant nodules. Category 3 in the Bethesda System, titled “Atypia of Undetermined Significance or Follicular Lesion of Undetermined Significance” (AUS/FLUS), poses particular uncertainty, as it indicates cellular abnormalities that are not definitive for malignancy. This classification requires further investigation to determine the risk of malignancy and guide appropriate clinical management. Various factors, such as the patient’s age, nodule size, and ultrasound characteristics, play crucial roles in assessing the risk of malignancy in these cases [12]. Additionally, the molecular testing of FNAC material has emerged as a valuable tool in stratifying the risk of malignancy in thyroid nodules categorized as AUS/FLUS, providing deeper insights into their biological behaviors and aiding in clinical decision making [13,14]. However, some experts argue that the reliance on molecular testing for the risk stratification of AUS/FLUS nodules may lead to overdiagnosis and overtreatment. They suggest that genetic alterations within the nodules do not always directly translate to an increased risk of malignancy, and there is a potential for unnecessary interventions based solely on molecular test results. Critics propose that a more cautious approach should be taken when interpreting molecular test findings in order to avoid causing unnecessary harm to patients through aggressive treatments [15,16]. Furthermore, the integration of clinical, radiological, and molecular data has enhanced the precision of risk stratification, allowing for a more personalized approach to the management of Bethesda category III thyroid nodules. As a result, clinicians are better equipped to recommend appropriate clinical interventions, including close monitoring, repeat fine needle aspiration, or surgical excision, based on the individualized risk assessment of AUS/FLUS nodules [17]. Nevertheless, there are controversial data about the risk of malignancies, recurrence, and the clinical management of nodules in Bethesda category III, as the reported risks of malignancy vary significantly from 6 to 52% [18,19,20].

This research examines the risk for malignancy in thyroid nodule FNAC samples categorized as Bethesda III. It is essential to comprehend the risk associated with this category in order to guide subsequent decisions and management, specifically regarding the necessity for repeated FNAC or surgical intervention.

## 2. Materials and Methods

Particularly for patients enduring thyroid surgery, the Burjeel Endocrine Surgery Centre has established a prospectively maintained surgical database. Data from 1038 consecutive patients who had bilateral or unilateral thyroidectomy by a single endocrine surgeon (I.H.) at a tertiary hospital were retrospectively analyzed between January 2020 and March 2024. Our study included 670 out of 1038 patients who underwent thyroid surgery and had preoperative FNAC of thyroid nodules with at least 30 days of follow-up. We previously described the standard operating room (OR) procedures for thyroid surgery at our institution, including patient placement on the OR table, anesthesia selection, equipment setup, and neuromonitoring [21].

### 2.1. FNAC Technique

**Figure 1:**
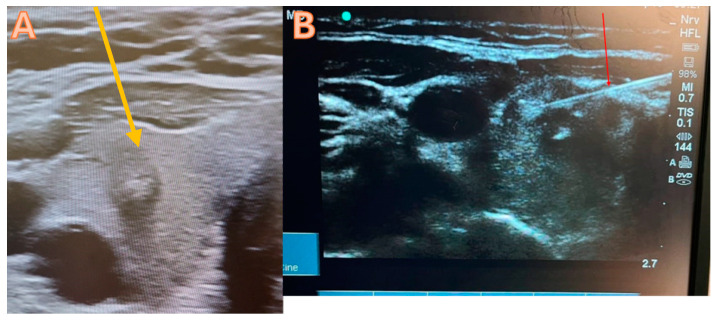
Staining of the thyroid FNAC sample. (**A**) The high-resolution ultrasound yellow arrow suggests that there is a potentially malignant tumor in the right lobe of the thyroid, categorized as Tirad 4. (**B**) FNAC trans isthmic; the red arrow denotes the accurate placement of the needle into the nodule.

Typically, thyroid FNAC is carried out under local anesthesia in an outpatient setting with ultrasound guidance. In order to complete the procedure, fasting is not required. A thorough explanation of the procedure will be given to the patient by using pictures and video, and then a consent form will be obtained. The procedure starts with the use of a high-resolution ultrasound to determine the dominant nodule that requires FNAC (Figure 1A). After cleaning and sterilizing the area, 1 mL of lidocaine (2%) is administered to the intended site. The next step involves the trans-isthmic insertion of a 21G needle into the thyroid nodules under ultrasound guidance, requiring multiple passes to ensure proper material delivery (Figure 1B). Next, the needle is withdrawn and the aspirate is equally distributed among the slides. Before the slides dry, they are immersed in a 95% alcohol container for 30 min to guarantee sufficient fixation. Afterwards, the pathology lab will obtain the slides and proceed with their analysis. At the injection site, a plaster and an Eis pack will be applied. The patient will be monitored for thirty minutes before being discharged.

Next, 95% ethanol-fixed smears are stained with Papanicolaou with the Dako Cover Staining System (Dako SP30, Dako Agilent, Santa Clara, CA, USA). Fixation prepares the sample from different sites of the body for the purpose of preserving and maintaining the existing form and structure of all constituent elements. A delay in fixation will result in air-dried artefact changes with a loss of cellular details. However, nuclear details are well visualized in Papanicolaou-stained smears at 200 or 400× magnification.

Staining of formalin-fixed paraffin-embedded (FFPE) sections of surgical specimens are accessioned and macroscopically described by anatomic pathologists. The samples were paraffin-embedded according to standard techniques after 24 to 48 h of fixation in 10% neutral buffered formalin. Specimens sampled in cassettes were dehydrated with various grades of alcohol, cleared by xylene, and impregnated with paraffin wax with the aid of a vacuum infiltration processor (Tissue Tek VIP 6, Sakura, Torrance, CA, USA). Samples were embedded to prepare them for microtomy (Tissue-Tek TEC 5, Sakura, USA). Sections 3 to 5 μm in thickness were cut from FFPE using an Accu-Cut^®^ SRM™ 200 Rotary Microtome (Sakura, USA), and the sections were mounted onto glass slides. Sections were baked before being subjected to staining. An automated hematoxylin and eosin (H&E) staining machine was used (VENTANA HE 600 system, Roche, Indianapolis, IN, USA). The HE600 system deparaffinized the sections using xylene and was rehydrated with various grades of alcohol. Primary dye Harris hematoxylin was used for nuclear staining, differentiated by acidified alcohol and blued by alkaline water. Sections were subjected to the secondary dye Eosin Y to stain the cytoplasm and extracellular matrix. The sections were again dehydrated with various graded alcohols, cleared with xylene, and coverslipped using a mounting medium. Thyroid samples were stained by immunohistochemistry using Dako Link48/PT Link (Dako Agilent, USA) if required. For further research, all stained sections were examined using a light microscope (Olympus microscope BX 46F, Hachiojo, Japan).

### 2.2. Data Acquisition

The surgical database combines preoperative, intraoperative, and postoperative clinicopathological criteria that are particularly relevant to the progression of thyroid cancer. Standard patient characteristics, laboratory findings, and imaging investigations are also included in this resource.

Upon completing the microscopic review, the results were evaluated and double-checked by at least one additional qualified pathologist at our institution. They described the Bethesda category based on typical cytomorphologic characteristics, including architectural atypia, which is characterized by the presence of cellular clusters exhibiting pale and enlarged nuclei, as well as nuclear crowding and overlapping. Also, a histopathological assessment of a surgical specimen of papillary thyroid cancer was performed. The diagnostic criteria for this condition are as follows: Papillary architecture: tumor cells protrude into thyroid follicles in a papillary growth pattern. The formations are called “papillae”. Nuclear features: PTC cells display nuclear expansion, clearing (ground-glass appearance), grooves, and pseudo inclusions. The “Orphan Annie eye” nuclei of PTC cells are widely mentioned. Psammoma bodies: Tumor stroma calcifications are concentric and lamellated. Classic PTC has psammoma bodies.

### 2.3. Statistical Analysis

A statistical analysis was conducted using IBM SPSS version 29 (SPSS Inc., Chicago, IL, USA). The parametric data are shown as the average value together with the measure of variability, which is the standard deviation. On the other hand, the non-parametric data are represented by the middle value (median rank) along with the range between the first and third quartiles (interquartile range). The univariate analysis included Student’s t-test and the Mann–Whitney U-test for continuous variables and Fisher’s exact test for categorical data. A *p*-value was considered statistically significant if it was below 0.05.

## 3. Results

From January 2020 to March 2024, a grand total of 1038 patients who had thyroidectomy for a thyroid condition were identified. A total of 670 patients had preoperative FNAC, which was included in the final analysis. In this study, the ratio of females to males was 137:533 in the entire group, and the median age was 43 years (SD 11.8). However, 47.5% of the surgical specimens were malignant. The younger patients had a more malignant surgical histology than the benign ones. Patients who were diagnosed with benign disease had an average age of 44.7 years, whereas those diagnosed with malignancy had an average age of 41.1 years (Anova test, *p* = 0.0001). The histopathology features showed a trend towards larger tumor foci in male patients (average 13.4 mm) when compared to females (average 11.7 mm). However, while female patients had significantly more less invasive tumors (chi-square test, *p* = 0.0001) and poor prognostic factors, and bilateral multifocality did not differ between the genders (chi-square test, *p* = 0.052). Additional characteristics related to the comparison between the two genders are shown in Table 1 and Table 2.

### 3.1. Comparison with Other Bethesda Categories

**Figure 2:**
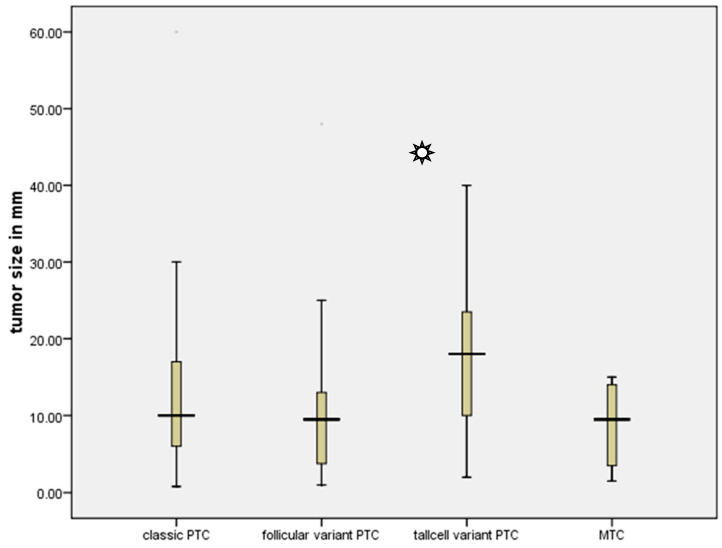
Tumor size in relation to different types of Thyroid cancer. 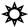 indicates *p* = 0.049 in Kruskal–Wallis test.

The prevalence of cancer in Bethesda III nodules was comparable to that of Bethesda IV, with rates of 33.5% and 33.8%, respectively. However, the incidence of cancer in Bethesda III nodules was lower than that in Bethesda V nodules, which had a malignancy rate of 98.3%. It is worth noting that the risk of malignancy in Bethesda VI nodules is precisely the same as that in Bethesda V nodules. However, the risk of malignancy for Bethesda I was 17.6%, which is comparable to the risk of malignancy in the Bethesda II group, which is 18.3% (Table 3). In addition, the complete group consisted of 201 instances of classic papillary thyroid cancer, 52 cases of follicular variation of papillary thyroid cancer, 19 cases of tall cell variant, and 12 cases of medullary thyroid cancer. Nevertheless, the tall cell variant exhibited a significantly larger mean diameter of 17.5 mm in comparison to all other tumors (Kruskal–Wallis test, *p* = 0.49) (Figure 2).

### 3.2. The impact of Hurthle Cells in the FNAC Sample

Hurthle cells were prominently present in the fine needle aspiration samples from 122 out of 670 patients, representing 18.2% of the total. The final surgical histology revealed 44 malignant cases (36%) and 78 benign cases (64%) (Fisher’s exact test, sample size = 0.002). A Hurthle cell predominate nodule was discovered in 53 out of 170 patients (31.2%) in the Bethesda category III subgroup. Out of these 53 instances, only 18 (34%) were identified to have a malignant nodule, whereas 35 cases (64%) were benign in the surgical histology (chi-square test, *p* = 0.0001). On the other hand, out of the 117 patients who had a hurthle cell-negative FNAC sample, 39 instances (33.3%) were found to be malignant, whereas 78 cases (66.7%) were found to be benign in the final surgical histopathology (chi-square test, *p* = 0.0001).

## 4. Discussion

Thyroid carcinoma is the most common kind of cancer affecting the endocrine system, and its occurrence is on the rise globally. Thyroid cancer is three times more common in women than in men in the United Arab Emirates. The evaluation of a thyroid gland lesion requires a thorough examination using clinical, radiographic, cytological, and histological techniques. The early detection of thyroid tumors is crucial, as new information suggests that more aggressive forms have emerged in the wake of the COVID-19 pandemic [4,5]. Several classification strategies have been developed to reduce discrepancies among observers while reporting thyroid cytology. The Bethesda Reporting System of Thyroid Cytopathology was established in 2009 and has gained widespread acceptance in the United States of America, the Arabian Gulf, and several other regions of the globe. It has experienced various adjustments throughout the years [22,23].

Thyroid nodules classified as Bethesda III, especially Atypia of Undetermined Significance or Follicular Lesion of Undetermined Significance (AUS/FLUS), provide unique challenges in medical management. The precise risk of malignancy for this category remains undetermined due to the presence of several nodules categorized as AUS/FLUS that have not undergone verification by surgical pathology. In 2009, the first edition of TBRTCS projected that the probability of malignancy for Bethesda category III was between 5 and 15% [22]. However, the third edition of TBSRTC released in 2023 revealed a higher risk of malignancy at 22% [23]. Other researchers in recent studies in the literature have even reported a higher risk of malignancy for Bethesda II and Bethesda category III. For instance, Inabnet et al. reported a large cohort of 21764 patients who underwent thyroidectomy in 314 institutions across 22 countries. The study compared the findings of FNAC with surgical specimens. In their analysis, Bethesda category II was associated with an approximately 13% risk of malignancy, while Bethesda category III was associated with a 32% risk. A subsequent analysis of the same cohort revealed that male patients categorized as Bethesda III aged 36–40 had a 56% increased risk of malignancy [20].

In our study, the Bethesda II category of patients constituted 26.9%, followed by Bethesda III, constituting 25.4%. Our investigation showed that both Bethesda categories II and III had a malignancy rate that exceeded the limit specified by the first and third edition of the Bethesda System. This study’s findings indicate that the higher malignancy incidence is closely linked to the presence of referral and selection bias since the research was conducted at a tertiary care center where patients were referred due to a higher likelihood of malignancy.

The uncertainty surrounding the potential for malignancy in these nodules underscores the importance of an accurate risk assessment and appropriate management strategies. In this context, the present study aimed to investigate the risk of malignancy of Bethesda category III in a large patient cohort operated at a high-volume endocrine surgery center in the United Arab Emirates. Based on the findings of the current study, the overall rate of malignancy was estimated to be 47.5%. Additionally, for nodules classified as Bethesda category III, the malignancy rate was 33.5%. The prevalence of our data is similar to that of the research conducted by Thakkar et al. [24], which suggested a range of 26.6%–37.8% for Bethesda category III. However, Alhassan et al., also from the Gulf region, only reportedin 137 patients who had underwent thyroid surgery with concomitant preoperative FNACand found a malignancy rate of 55.6% in the Bethesda III category [25]. Ali Khalil et al. reported a malignancy rate of 20% for indetermined nodules in a previous study in the UAE involving 584 patients [26]. The discrepancy in the malignancy rate of an indeterminate nodule between Ali Khalil’s published study and the current study may be attributed to the utilization of distinct classification systems. Ali Khalil’s study employed the United Kingdom Royal College of Pathologists Thy Terminology, whereas the current study adopted the Bethesda classification system. Their limited number of surgical cases may also account for the difference. Specifically, only 30 patients in their study received surgical resection for an indeterminate nodule, while our analysis included 170 individuals who underwent surgery for the same reason. A further study conducted by Fatima et al. in a related geographical area yielded comparable results for Bethesda category III. Out of the 104 resection specimens with preoperative FNAC classified as Bethesda III in their study, 72 (69.2%) were determined to be benign, whereas 32 instances (30.7%) were diagnosed as malignant [27]. However, some European research, on the other hand, revealed a malignancy risk of 15% for Bethesda category III when compared to surgical histopathology, which is in line with the original TBSRTC data [28].

In contrast to previous research findings, our study did not demonstrate a gender bias towards malignancy in Bethesda category III [29]. This is because malignant nodules accounted for one-third of the total group in both males and females. However, malignant nodules in Bethesda category III were more prevalent in younger individuals with a mean age of 41 years, while benign nodules were detected at an average age of 44 years. This finding might confirm the finding published by Walters et al., who observed in a cohort of a comparable size to ours that the likelihood of malignancy for Bethesda III is 38.3%, with a younger age being the only predictor that showed statistical significance in terms of an increased risk of malignancy [30].

Another noteworthy finding from our analysis is the impact of an FNAC sample with a significant amount of hurthle cells on estimating the risk of malignancy in a suspicious nodule. The reason for this is that FNAC samples with prominent hurthle cell features remain challenging for review. In our investigation, 18.2% of the samples had a hurthle cell predominance rate, meaning that over 50% of the cells in the FNAC sample were hurthle cells. These proportions are not markedly different from those reported by other investigators [31,32]. When compared to surgical pathology, we found only one-third of the predominant hurthle cell nodules, as shown in the FNAC sample, to be malignant. This is in line with recent reports by Ren et al., who found that malignancy risk is not impacted by hurthle cell-predominant FNAC [33]. This is particularly important in order to avoid the over-interpretation of hurthle cell lesions and facilitate appropriate patient management.

Lastly, our analysis revealed that the malignancy rate for nodules categorized as Bethesda category II was 18.3%, a much higher rate than that which was published in the third edition of Bethesda, which maintained a 4% risk of malignancy for Bethesda category II nodules [23]. However, a recent study conducted by Mulita et al. observed that the malignancy risk for the Bethesda II category is even lower at 1.58% [34]. In contrast, a study by Muri et al. involving 5030 patients showed a malignancy risk of 9% [35], which is similar to the previously mentioned study from the European and American registry that reported a malignancy risk of 12.7% for the Bethesda II category [20]. The underreporting of clinical variables that influence the decision to proceed with surgical intervention despite a benign cytological diagnosis may account for the discrepancy in the risk of malignancy associated with category II specimens between our study and others. Thyroid dysfunction, nodule size, local compression, ultrasound features, family history, patient preference, and radiation exposure history are a few more risk factors that need to be considered when evaluating the likelihood of malignancy and choosing a surgical procedure in this category.

There are several limitations inherent in our study. The main constraint is the lack of available data pertaining to patients who underwent molecular testing using the FNAC sample. The main hindrance to acquiring this vital measure was the absence of insurance coverage and the unavailability of the technology in the UAE, necessitating the dispatch of samples and further augmenting the expense. Furthermore, the Bethesda III group remains a small cohort, even with 170 patients, limiting our ability to draw more definitive conclusions. We attribute the limited relevance of our results to the retrospective nature of our investigation, which was conducted at a single site. To assess our results, it is necessary to conduct prospective studies, preferably with larger sample sizes from diverse geographies and with a specific emphasis on genetic and biological research.

## 5. Conclusions

Our study results indicate that high-volume centers may have a greater incidence of malignant thyroid nodules in the final surgical pathology since our endocrine surgery center detected malignancy in 47.5% of the patients that underwent thyroidectomy. Furthermore, Bethesda categories I-III are associated with a heightened risk of malignancy compared to the third edition of TBSRTC, particularly among younger patients. Furthermore, our data indicate a notable gender disparity in malignancy risk within Bethesda category II, with female patients exhibiting a higher susceptibility compared to their male counterparts. This emphasizes the necessity of individualized risk assessments and patient counselling, taking into account gender-specific factors when determining the optimal course of action.

In summary, our study underscores the evolving landscape of thyroid nodule risk stratification in the Arabian Gulf region and highlights the imperative for nuanced, patient-centered approaches to optimize diagnostic accuracy and therapeutic outcomes. Further research is warranted to elucidate the underlying factors contributing to these observed trends and to refine risk assessment strategies tailored to the unique characteristics of the population under study.

## Figures and Tables

**Table 1:** A comparison of the clinical and pathological features between the genders.

	Male	Female	Total	*p*-Value
Mean age in years	45.1 *	42.5		0.02
Hurthle cell predominant FNAC (*n*)	17 (12.4%) †	105 (19.7%)	122 (18.2%)	0.049
Malignant/benign surgical specimen	68/69	250/283	318/352	0.317
Median tumor size in mm	13.4	11.7		0.217
Multifocality and bilateral (*n*)	39 (59.1%)	194 (74%)	233 (71%)	0.052
Noninvasively tumors (*n*)	45 (61.6%) †	249 (81.9%)	294 (78%)	0.0001
Lobectomy/total thyroidectomy	41/96	130/403	171/499	0.055
Resection time in minutes	95.6 *	72.3		0.001

† denotes a statistically significant difference between the groups, as determined using a chi-square test. * denotes a statistically significant difference between the groups, as determined using a *t*-test. (*n*) is the absolute number of cases.

**Table 2:** A comparison of Bethesda categories of FNAC samples across genders. The results indicate that there is no difference in the distribution of Bethesda categories.

Bethesda Category		Male	Female	Total
Bethesda I (unsatisfactory)	Count	8	26	34
	% within genders	5.80%	4.870%	5.0%
Bethesda II (benign)	Count	29	151	180
	% within genders	21.20%	28.33%	26.90%
Bethesda III (AUS)	Count	33	137	170
	% within genders	24.10%	25.70%	25.40%
Bethesda IV (follicular neoplasm)	Count	15	62	77
	% within genders	10.90%	11.63%	11.50%
Bethesda V (suspicious for malignancy)	Count	32	96	128
	% within genders	23.40%	18.00%	19.10%
Bethesda VI (malignant)	Count	20	61	81
	% within genders	14.60%	11.44%	12.10%
Total	Count	137	533	670
	% within genders	100.00%	100.00%	100.00%

**Table 3:** Distribution of Bethesda categories in FNAC samples across surgical pathology entities, presenting actual numbers and percentage distribution.

			Surgical Histopatholgy		Total 18.3%
			Malignant Nodule	Benign Nodule	
FNAC Categories	Bethesda I (unsatisfactory)	Count	6	28	34
		% within FNAC categories	17.60%	82.40%	100.00%
	Bethesda II (benign)	Count	33	147	180
		% within FNAC categories	18.30%	81.70%	100.00%
	Bethesda III (AUS)	Count	57	113	170
		% within FNAC categories	33.50%	66.50%	100.00%
	Bethesda IV (follicular neoplasm)	Count	26	51	77
		% within FNAC categories	33.80%	66.20%	100.00%
	Bethesda V (suspicious for malignancy)	Count	120	8	128
		% within FNAC categories	93.80%	6.20%	100.00%
	Bethesda VI (malignant)	Count,	76	5	81
		% within FNAC categories	93.80%	6.20%	100.00%
Total		Count	318	352	670
		% within FNAC categories	47.50%	52.50%	100.00%

## Data Availability

The data that support the results of this study can be requested from the corresponding author.

## Abbreviations

PTC	Papillary Thyroid Cancer
FNAC	Fine Needle Aspiration Cytology
TBSRTC	The Bethesda System for Reporting Thyroid Cytopathology
COVID-19	Coronavirus Disease 2019
TC	Thyroid Cancer
AUS	Atypia of Undetermined Significance
FLUS	Follicular Lesion of Undetermined Significance
FFPE	Formalin-Fixed Paraffin-Embedded
HE	Hematoxylin and Eosin
